# Optimizing the Control of Anteromedial Rotatory Knee Instability: A Biomechanical Validation of Different Anteromedial Reconstruction Techniques

**DOI:** 10.1177/03635465251339820

**Published:** 2025-05-15

**Authors:** Florian Gellhaus, James R. Robinson, Martin Lind, Adrian Deichsel, Matthias Klimek, Nina Backheuer, Michael J. Raschke, Andreas Seekamp, Peter Behrendt, Christoph Kittl

**Affiliations:** * Department of Orthopedic and Trauma Surgery, University Medical Center Schleswig-Holstein, Campus Kiel, Kiel, Germany; † Department of Anatomy, Christian-Albrechts-University Kiel, Kiel, Germany; ‡ Knee Specialists, Bristol, UK; § Department of Orthopedics, Aarhus University Hospital, Aarhus, Denmark; ‖ Department of Trauma, Hand and Reconstructive Surgery, University of Münster, Münster, Germany; ¶ Orthopedic Surgery Kiel, Kiel, Germany; Investigation performed at the Department of Trauma, Hand and Reconstructive Surgery, University Hospital Münster, Münster, Germany

**Keywords:** MCL, biomechanics, reconstruction, AMRI, ACL

## Abstract

**Background:**

Anteromedial rotatory instability (AMRI) can result from combined injury to the anterior cruciate ligament (ACL) and medial collateral ligament (MCL) complex (superficial and deep [sMCL and dMCL]).

**Hypothesis:**

Adding an oblique anteromedial (AM) limb to an sMCL reconstruction improves the control of AMRI.

**Study Design:**

Controlled laboratory study.

**Methods:**

A 6 degrees of freedom robotic setup simulated clinical laxity in 9 unpaired knees under the following tests: 5-N·m external rotation (ER), 8-N·m valgus rotation (VR), and AM drawer (combined 89-N anterior tibial translation and 5-N·m ER). Knees were tested intact after cutting the sMCL and dMCL and after 5 different reconstructions: modified Lind, short sMCL, and sMCL with the addition of an AM graft limb with 3 different obliquities.

**Results:**

After short sMCL reconstruction, AM drawer and ER laxity were not significantly different from either the MCL-deficient state or the intact state. VR was reduced from the MCL-deficient state between 0° and 60° of flexion but not at 90°. For combined sMCL + AM reconstructions, VR was reduced as compared with the MCL-deficient state at all flexion angles. AM drawer laxity and ER laxity were also reduced, similar to the intact state, except at 30° where, for the more oblique T1 and T2 AM reconstructions, laxity was less than in the intact state. The modified Lind reconstruction reduced AM drawer and ER laxity from the MCL-deficient state to values similar to the intact state at all flexion angles. VR laxity was also reduced at all flexion angles, similar to the intact knee at 0° to 30°. However, it was not as good at restraining AM drawer and ER when compared with the sMCL reconstructions with more oblique AM limbs.

**Conclusion:**

AMRI appears to be better restrained by adding an oblique AM graft limb to an sMCL reconstruction, replicating the function of the sMCL and dMCL in a cadaveric model. The tibial attachment of the AM limb should be anterior to the sMCL, but its precise attachment on the tibia is less important. This offers surgical flexibility, which may be helpful in avoiding anterior cruciate ligament tibial tunnel coalition. The femoral attachment on the posterior medial epicondyle is critical to optimize graft isometry.

**Clinical Relevance:**

Adding an AM limb to a medial reconstruction optimizes the control of AMRI at time zero. The tibial attachment site is less critical, offering surgical flexibility.

Injuries to the medial collateral ligament (MCL) complex are common and occur concomitantly in up to 77.5% of anterior cruciate ligament (ACL) injuries.^17,23,40,54^ There is consensus that the treatment of choice for partial MCL injuries combined with ACL rupture is a period of nonoperative management in a range of motion brace before delayed isolated ACL reconstruction, with many surgeons advocating a similar approach for complete medial-sided injuries combined with ACL rupture.^24^ Early MCL reconstruction and/or repair is usually advocated only for multiligamentous injuries involving 3 ligaments, tibial avulsions of the superficial MCL (sMCL), or femoral-sided bony avulsions.^16^ However, residual medial laxity after nonoperative treatment^17,32,38^ has been shown to negatively affect the outcome of ACL reconstruction with increased rates of graft failure.^2,3,6,49,54^ Yet the results of combined ACL reconstruction and MCL repair and/or reconstruction are not as good^38,50,51^ as those for patients who have had isolated ACL reconstruction.^32^ This may be a result of injuries involving 2 ligaments inherently leading to poorer outcomes, or it might suggest that current MCL techniques are suboptimal for combined ACL/MCL procedures.

MCL injuries sustained with ACL rupture frequently involve the sMCL and deep MCL (dMCL).^17^ The sMCL is the primary restraint to valgus rotation (VR), but it is also important in restraining anteromedial rotatory instability (AMRI),^4,7,28,53^ with the dMCL acting to restrain tibial external rotation (ER)^7^ and anterior tibial translation (ATT).^53^ Commonly used MCL reconstruction techniques typically address the sMCL and the posterior oblique ligament (POL).^37,39,44^ The POL fibers are, however, poorly aligned to control tibial ER and ATT. Recent studies suggest that reconstruction reproducing the sMCL and POL suboptimally control AMRI.^4,28^ The POL is an important restraint to VR and internal rotation (IR) in full knee extension and should be addressed if medial laxity is present in the fully extended knee.^28^

In an effort to better restrain AMRI, surgical reconstructions have been developed to reproduce the function of the dMCL and sMCL.^1,5,8,26,31^ An obliquely oriented anteromedial (AM) reconstruction, mimicking the function of the dMCL, has been suggested to improve the control of ATT and ER.^12,13^ There are, however, few data in the literature comparing the efficacy of the different reconstruction techniques in restoring native kinematics and how different AM graft orientations act to restrain AMRI.

We hypothesized that AMRI would be better controlled by an sMCL reconstruction combined with an anteriorly oriented AM reconstruction graft, replicating the function of the anterior fibers of the dMCL, as compared with an isolated sMCL reconstruction graft.

## Methods

Nine unpaired fresh-frozen human cadaveric knees were used in this study (mean age, 77.4 years; range, 67-92 years; 3 female, 6 male). The specimens were evaluated to ensure that there was no fixed flexion deformity, no degenerative joint disease, and no history of previous injury. The study was conducted according to the guidelines of the Declaration of Helsinki and was approved by the institutional review board (University of Münster; reference 2023-481-f-S). The specimens were dissected and tested by an orthopaedic resident with a supervising senior investigator. After testing, the specimens were further dissected to ensure the integrity of the ACL, menisci, and posterior cruciate ligament and to confirm the absence of any existing advanced cartilage erosions.

### Specimen Preparation

Specimens were stored at −20°C and thawed for 24 hours at room temperature before dissecting and testing. The tibia and femur were transected 20 cm from the joint line. The skin and subcutaneous tissue were removed, but the superficial fascia and the musculature were left intact. The fibula was cut 10 cm distal to the proximal tibiofibular joint and secured to the tibia in its anatomic position with a 3.5-mm positioning screw.^46^ The cut ends of the femur and tibia were secured in cylindrical aluminum tubes by epoxy resin. The tibia was mounted so that its longitudinal axis was oriented at 90° to the transepicondylar epicondyle axis of the femur with the knee in extension. Before testing, the gracilis and sartorius tendons were removed. Muscle was stripped from the semitendinosus (ST) tendon proximally and the tendon followed to the pes anserinus distally. The posterior part of the tibial insertion was carefully released to expose the tibial attachment of the sMCL, taking care to leaving the anterior ST insertion intact and the tendon still attached for subsequent use in the modified Lind reconstruction.

### Testing Setup

A 6 degrees of freedom industrial robot (KR 60-3; KUKA Robotics) equipped with a force-torque sensor (Theta; ATI Industrial Automation) was used for biomechanical testing as previously described.^8,19,53^ This system allowed for repeatability of motion within ±0.06 mm and accuracies of ±0.25 N and ±0.05 N·m for forces and torques.^8,19,53^ Testing was conducted in customized software designed for musculoskeletal robotic simulation (SimVitro; Cleveland Clinic BioRobotics Lab). A precise measuring arm (±0.05 mm, Absolute Arm 8320-7; Hexagon Metrology GmbH) was used to digitize anatomic landmarks and thus define the knee coordinate system based on the description by Grood and Suntay.^22^ The differences in rotations (degrees) and translations (millimeters) were calculated for each step of the testing protocol.

### Biomechanical Testing

To minimize tissue hysteresis, the specimens were flexed and extended 10 times before testing.^41^ The tibial cylinder was then fixed into the stationary base unit, and the femoral cylinder fixed to the manipulator arm of the robot. The neutral starting position of the knee was defined by minimizing forces (<1 N) and torques (<0.5 N·m) at full extension as in previous studies.^8,34^ The passive flexion-extension path between 0° and 90° of flexion was established for each knee. To ensure that contact between tibia and femur was maintained, 50 N of axial compression was applied. The neutral position was recorded at 0°, 30°, 60°, and 90° of knee flexion and tibiofemoral laxity measured in response to simulated clinical laxity tests performed for each testing state. The following tests were applied using the robotic setup in force control mode (ie, measuring movement in response to a given force/torque)^4,8,13^:

Anteromedial drawer test: 89 N of anterior tibial force in 5 N·m of ER, simulating the clinical Slocum and Larson test^48^
External rotation: 5 N·m
Internal rotation: 5 N·m
Valgus rotation: 8 N·m

For the AM drawer test, ATT was quantified as the change in distance between the midpoint of the tibial plateau and the midpoint between the femoral condyles.

### Cutting Protocol

After the knee was tested in its intact state, the sMCL and dMCL were cut. Care was taken not to damage the POL and the AM retinaculum. The sMCL was sharply dissected from its distal tibial attachment, followed proximally and released from its proximal tibial attachment, and then removed completely by sharp dissection from its proximal attachment on the medial femoral epicondyle. The meniscofemoral and meniscotibial portions of the dMCL were transected. To ensure complete dMCL insufficiency, the femoral attachment distal to the medial epicondyle was removed.

### Medial Reconstructions

**Figure 1.**
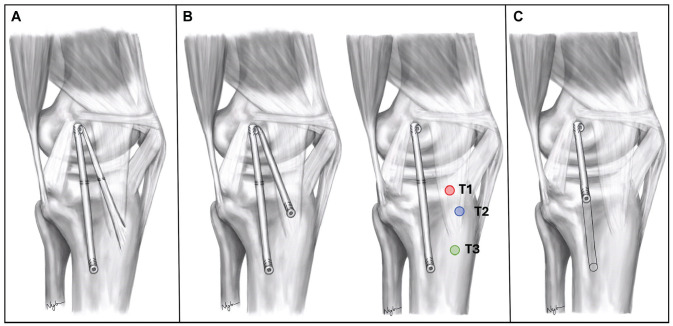
Illustration of tested reconstructions. (A) Modified Lind technique: semitendinosus tendon left attached to the tibia and to the femur in the posterior part of the medial epicondyle, then fixed distally at the tibial attachment of the sMCL. Additional suture anchors were used to fix both limbs of the graft 1.5 cm below the joint line. (B) Single-bundle sMCL reconstruction with 3 versions of an additional AM reconstruction (sMCL + AM) using different tibial attachments: T1, the tibial attachment of the anterior fibers of the native deep medial collateral ligament; T2, a point 2 cm posterior to the anterior cortex and 2 cm distal to the joint line; T3, the distal insertion site of the semitendinosus tendon. (C) A short sMCL reconstruction: a single-bundle reconstruction from the femoral sMCL attachment to a point 1.5 cm below joint line along the course of the native sMCL. AM, anteromedial; sMCL, superficial medial collateral ligament.

After each knee was tested in the intact and sectioned state, 5 medial reconstructions (Figure 1) were performed in a semirandomized order: (1) a modified Lind technique with an AM arm instead of the POL arm^15,39^; (2-4) a single-bundle sMCL reconstruction with AM reconstruction utilizing 3 tibial attachments (T1, T2, and T3) for the AM reconstruction; and (5) a short sMCL reconstruction. We chose to test the modified Lind reconstruction as a relatively straightforward method of achieving a double-bundle AM reconstruction utilizing the native attachment of the ST tendon as fixation for the AM limb. For the double-bundle reconstructions using different tibial attachments for the AM limb, we standardized the sMCL graft constructs using similar tibial fixation to that described by LaPrade and Wijdicks.^37^ A short sMCL construct was also tested, as this has been proposed to control AMRI.^12,47^ We did not test an isolated oblique AM graft, as the obliquity of this construct is likely to inadequately restrain VR laxity.^13^

First, a modified Lind technique was performed.^39,52^ The ST tendon was left attached to the tibia, and the tendon led to the femoral attachment, forming the AM limb of the reconstruction. The femoral attachment position was selected in the posterior third of the anatomic sMCL attachment on the medial epicondyle. The tibial attachment of the sMCL limb of the reconstruction was placed in the center of the native sMCL tibial attachment. These positions were selected according to previous length change pattern studies^9,36^ and have been shown to yield a low strain range across knee flexion. A K-wire was inserted into each attachment site. Graft length change behavior was checked using a thread between the 2 K-wires to confirm minimal length change and that the sMCL arm would be slightly tighter in extension than in flexion. Length changes of the AM limb were also tested using the ST tendon wrapped around the femoral K-wire to ensure near isometry of the AM limb.^9,36^ The femoral K-wire was then overdrilled to the diameter of the doubled ST tendon. The tibial K-wire at the center of the sMCL tibial attachment was overdrilled to the diameter of the single-stranded ST tendon (all graft diameters measured between 4.5 and 5.5 mm). The doubled ST tendon was pulled 20 mm into the femoral tunnel using a looped polyester suture (FiberWire; Arthrex Inc) and fixed onto a custom-made tensioning device.^8,18^ The free end of the ST tendon, representing the sMCL limb of the reconstruction, was whipstitched with polyester suture, pulled into the tibial tunnel, and fixed there onto a second tensioning device. Both strands of the graft were tensioned via, first, the femoral loop suture and, second, the tibial sMCL limb, both to 60 N at 20° of knee flexion, using the custom-made tensioning devices. The proximal tibial sMCL attachment was replicated by a suture that was subsequently passed through the tibia 15 mm below the joint line and secured via a button on the lateral tibial cortex. A similar fixation, 15 mm below the joint line, was used for the AM limb of the graft.

After testing of the Lind reconstruction, the ST tendon was excised from its tibial insertion at the pes anserinus. This end was whipstitched with polyester suture. The sMCL reconstructions with 3 tibial AM reconstructions (sMCL + AM reconstructions) were then performed and tested in a randomized order. K-wires, demarcating the tibial attachment sites of the AM reconstructions, were inserted at the anterior part of the native dMCL tibial attachment (T1), a point 2 cm posterior to the anterior tibial cortex and 2 cm distal to the joint line (T2), and the distal insertion of the ST tendon (T3). For the sMCL limb of the reconstructions, the femoral and tibial tunnels from the modified Lind reconstruction were reused. Graft isometry was again confirmed between the femoral attachment and the AM tibial K-wire positions using the ST tendon wrapped over the wire. For these reconstructions, the femoral graft was fixed statically by a transosseous button, and the tibial limbs of the sMCL and AM were attached to custom-made tensioning devices.^8^ After overdrilling the AM K-wire to the diameter of the ST tendon, the free end of the graft was passed into the AM tibial tunnel and fixed onto the tensioning device. The sMCL and AM limbs of the graft construct were tensioned to 60 N with the knee at 20° of flexion and in neutral IR/ER.

For the short sMCL construct, the same femoral sMCL attachment site was used as for the other reconstruction techniques. The graft, attached to a cortical fixation button, was pulled into the femoral tunnel and the button fixed at the lateral cortex of the femur. The sMCL graft was then pulled proximally, and an interference screw was placed in the distal half of the femoral tunnel aperture to position the graft in the proximal 12-o’clock position in the tunnel. Isometric behavior was tested before the reconstruction was assessed. The tibial tunnel was created 15 mm below the joint line and in the line of the central fibers of the sMCL. This position was chosen to mimic the proximal sMCL tibial attachment. The free tibial end of the graft was passed into the tibial tunnel and fixed to a tensioning device at the lateral tibial cortex. Tensioning was performed at 20° of knee flexion in neutral rotation.

### Data and Statistical Analysis

The primary outcome measure was the ATT (millimeters) during the AM drawer test to evaluate the efficacy with which each reconstruction restrained AMRI. Secondary outcomes were axial tibial rotation (ER and IR; degrees) and VR (degrees). Data were processed in MATLAB (Version R2019a; MathWorks). Kinematic differences between the reconstructions were analyzed by a 2-factor repeated measures analysis of variance (2-way analysis of variance) with a post hoc Bonferroni correction for multiple comparisons (Prism Version 10; GraphPad Software Inc). Based on previous medial knee laxity research,^8,20,53^ a power analysis (G*Power Version 3.1.9.7; α = .05) determined that a sample size of 7 specimens would be necessary to detect a 2° and 2-mm change with a power of 0.88 and 95% confidence.

## Results

### AM Drawer Test: Simulated Slocum and Larson Test

**Figure 2.**
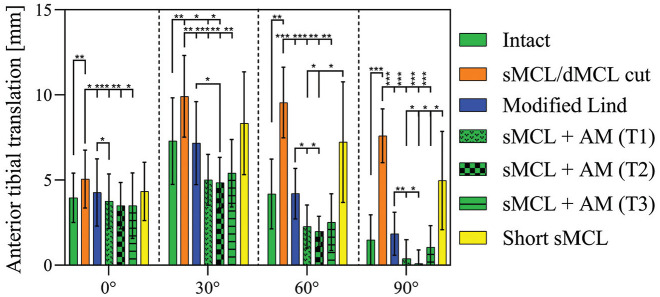
Anteromedial drawer test. Anterior tibial translation (millimeters) with application of an 89-N anterior drawer force, with the knee in 5 N·m of external rotation. Testing was performed in the intact knee after cutting of the sMCL and dMCL and after each medial reconstruction (n = 9). Data are presented as mean ± SD. *P < .05. **P < .01. ***P < .001. AM, anteromedial; dMCL, deep medial collateral ligament; sMCL, superficial medial collateral ligament.

Cutting the sMCL and dMCL increased ATT at all angles of knee flexion (*P*≤ .059; Figure 2): from 4 ± 1.5 mm (mean ± SD; intact knee) to 5 ± 1.7 mm (sMCL and dMCL cut) at 0° of flexion and from 1.5 ± 1.5 (intact knee) to 7.6 ± 1.6 mm (sMCL and dMCL cut) at 90° of flexion. After the modified Lind reconstruction, ATT laxity was significantly reduced as compared with the sMCL and dMCL cut state at all angles of knee flexion (*P*≤ .03) and was not significantly different from the intact state.

The sMCL + AM reconstructions, all performed similarly irrespective of the position of the tibial attachment of the AM limb (T1-T3), all resulted in a significant reduction of ATT as compared with the sMCL and dMCL sectioned state at all angles of knee flexion (*P*≤ .015). At 30° of flexion, ATT was decreased versus the intact knee with sMCL + AM T1 and T2 (*P*≤ .032). When compared with the modified Lind reconstruction, the sMCL + AM T1 was more effective in restraining ATT at 0° and 60° (*P*≤ .024) and at 90° (*P* = .008). There was no difference at 30° of flexion. The sMCL + AM T2 reduced ATT more than the modified Lind reconstruction at 30°, 60°, and 90° (0°, not significant; 30°-90°, *P*≤ .045). There was no difference in the effect on ATT between the modified Lind reconstruction and the sMCL + AM T3 reconstruction.

After short sMCL reconstruction, ATT was similar to the sMCL and dMCL cut state in all degrees of flexion but not significantly different from the native knee. The short sMCL reconstruction was significantly less potent at reducing ATT than the sMCL + AM T1 and T2 reconstructions at 60° of flexion (*P*≤ .04) and the sMCL + AM T1-T3 reconstructions at 90° of flexion (*P*≤ .036).

### External Rotation

**Figure 3.**
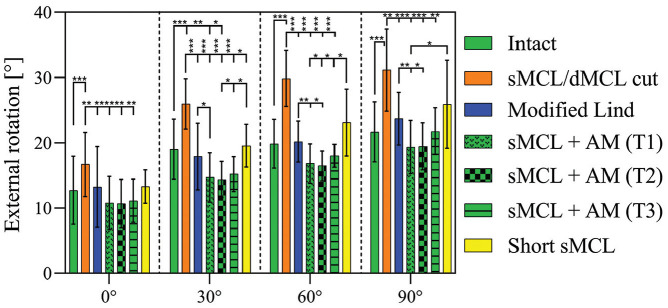
External rotation test. Tibial external rotation (degrees) with application of a 5-N·m external rotation torque in the intact knee after cutting of the sMCL and dMCL and after each medial reconstruction. Data are presented as mean ± SD. *P < .05. **P < .01. ***P < .001. AM, anteromedial; dMCL, deep medial collateral ligament; sMCL, superficial medial collateral ligament.

Cutting the sMCL and dMCL increased ER significantly (Figure 3) at all angles of knee flexion (*P* < .001): from 12.7°± 5.2° (intact knee) to 16.7°± 4.9° (sMCL and dMCL cut) at 0° of flexion and from 21.7°± 4.6° (intact knee) to 31.2°± 6.3° (sMCL and dMCL cut) at 90° of flexion. After the modified Lind reconstruction, ER laxity was reduced as compared with the sMCL- and dMCL-deficient state (*P*≤ .004) and was not significantly different from the intact state. The sMCL + AM reconstructions (T1-T3) also all reduced ER laxity at all knee flexion angles as compared with the sMCL- and dMCL-deficient state (*P*≤ .002). When compared with the modified Lind reconstruction, the sMCL+ AM T1 had a greater effect at reducing ER at 30°, 60°, and 90° (*P*≤ .026), and the sMCL + AM T2 was more effective than the modified Lind at 60° and 90° (*P*≤ .022). When compared with the sMCL and dMCL cut state, ER laxity after short sMCL reconstruction was not significantly different at 0°, 60°, and 90°, although there was a significant reduction at 30° (*P* = .038). However, the ER laxity after short sMCL reconstruction was not significantly different from the intact state at all angles of knee flexion. There was less reduction in ER laxity after short sMCL reconstruction versus sMCL + AM T1 at 60° and 90° of knee flexion (*P*≤ .038) and sMCL + AM T2 and T3 at 30° and 60° of knee flexion (*P*≤ .047).

### Valgus Rotation

**Figure 4.**
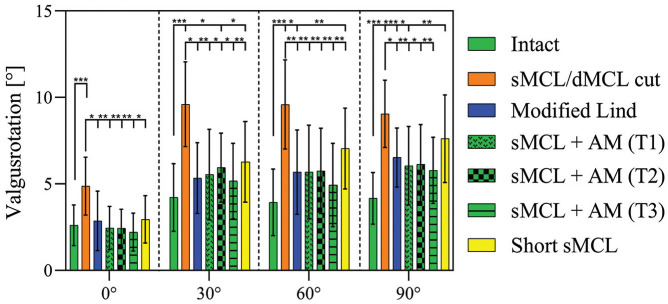
Valgus rotation test. Valgus rotation with an applied 8 N·m of valgus force in the intact knee after cutting of the sMCL and dMCL and after each medial reconstruction. Data are presented as mean ± SD. *P < .05. **P < .01. ***P < .001. AM, anteromedial; dMCL, deep medial collateral ligament; sMCL, superficial medial collateral ligament.

Cutting the sMCL and dMCL resulted in increased VR laxity (Figure 4) when compared with the intact state at all angles of knee flexion (*P* < .001) with an increase from 2.6°± 1.2° (intact knee) to 4.9°± 1.7° (sMCL and dMCL cut) at 0° of flexion and from 4.2°± 1.5° (intact knee) to 9.1°± 1.9° (sMCL and dMCL cut) at 90° of flexion. VR after modified Lind reconstruction was reduced at all degrees of knee flexion (*P*≤ .021) as compared with the sMCL and dMCL cut state. However, while VR after the modified Lind reconstruction did not differ from the intact state at 0° and 30°, it remained increased at 60° and 90° of knee flexion (*P*≤ .028).

The sMCL reconstructions with 3 AM reconstructions performed similarly in their effect on reducing VR laxity at all angles of knee flexion when compared with the sMCL and dMCL cut state. VR laxity was not significantly different from the intact state at all degrees of flexion, except for sMCL + AM T2 reconstruction at 30° (*P* = .023) and sMCL + AM T1 at 90° (*P* = .041)

After short sMCL reconstruction, VR was decreased when compared with the sMCL and dMCL cut state at 0° to 60° knee flexion (*P*≤ .011), but there was no significant difference at 90°. However, when compared with the intact state, VR remained increased at 30°, 60°, and 90° (*P*≤ .017) with no significant difference at 0° of flexion.

### Internal Rotation

**Figure 5.**
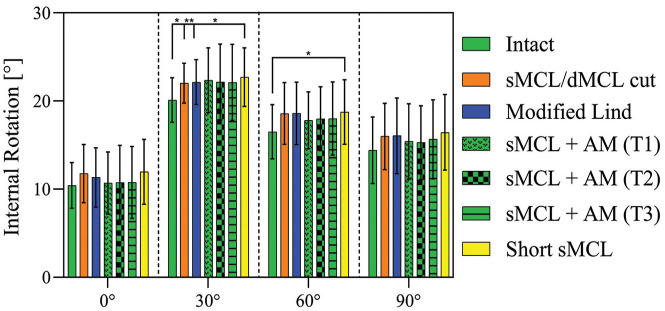
Internal rotation test. Internal rotation with an applied 5 N·m of internal rotation torque in the intact knee after cutting of the sMCL and dMCL and after each medial reconstruction. Data are presented as mean ± SD. *P < .05. **P < .01. AM, anteromedial; dMCL, deep medial collateral ligament; sMCL, superficial medial collateral ligament.

In 0° and 90° of flexion, no significant differences appeared between the native, sMCL, and dMCL sectioned and reconstructed states (Figure 5). In 30° of flexion, there was a significant increase of IR from the native knee (20.1°± 2.5°) toward (1) the sMCL and dMCL cut state of around 2° (22°± 2.3°; *P* = .014), (2) the modified Lind technique with an increase of around 2° (22.1°± 2.5°; *P* = .009), and (3) the short sMCL, also with an increase of around 2° (22.1°± 3.3°; *P* = .018). In 60° of knee flexion, there was a small but significant increase of around 2° between the short sMCL reconstruction and the native knee (native, 16.5°± 3.1°; short sMCL, 18.8°± 3.7°; *P* = .038). There were no significant differences between the reconstruction techniques. sMCL + AM reconstructions did not overconstrain IR when compared with the intact state.

## Discussion

The most important findings of this biomechanical study were that valgus laxity, ER laxity, and ATT laxity with AM drawer in the sMCL- and dMCL-deficient knee could be restored by a modified Lind reconstruction or a combined sMCL + AM reconstruction. The position of the tibial attachment of the AM reconstruction had little effect on efficacy. All reconstructions restored laxity to the native knee; however, the short sMCL reconstruction did not significantly differ from the deficient state at all angles of flexion. The combined sMCL + AM reconstructions were more potent at restraining ER and ATT in the sMCL- and dMCL-deficient state at some knee flexion angles as compared with the modified Lind or short sMCL reconstructions.

Several reconstruction techniques have been proposed to address AMRI,^1,5,8,12,13,26,31^ but few studies have biomechanically tested and compared different reconstructions. It has been suggested that an isolated single-bundle sMCL reconstruction may inadequately control AMRI and that the addition of an AM reconstruction mimicking the function of the dMCL may restore kinematics more closely to that of the intact knee.^8,42^ Others have recommended a short isometric sMCL construct,^12,13,47^ centered at the medial epicondyle but with a more proximal tibial attachment.

The complex functionality of the broad, flat sMCL and the underlying fan-shaped dMCL cannot be imitated by a single-bundle point-to-point sMCL reconstruction using the native sMCL attachment sites.^8,47^ Although Herbst et al^28^ demonstrated that the anterior-most fibers of the native sMCL are the most important for restraining AMRI, utilizing the anterior part of the sMCL femoral attachment site for an sMCL reconstruction leads to an adverse tensioning pattern, with the AM graft being looser in extension and tightening with flexion.^9,36^ Placing the sMCL femoral attachment site in the middle or posterior part of the native sMCL femoral attachment site gives a more favorable graft isometry.^9,36^ However, while beneficial for the control of VR throughout the range of knee flexion, the more posterior placement of the femoral attachment site replicates the posterior sMCL fibers, which are less effective in controlling AMRI.^28^ Yet, by adding an AM reconstruction with the posterior part of the native sMCL femoral attachment for the sMCL + AM reconstructions, optimal control of VR and AMRI could be achieved.^9,36^ We similarly found in this study that an sMCL + AM reconstruction reduced VR, ATT, and ER laxity in the sMCL- and dMCL-deficient knee throughout the range of knee flexion.

Shatrov et al^47^ recently reported that a short single-bundle sMCL reconstruction may be sufficient to control AMRI. The authors used an 8 mm–wide synthetic graft with a femoral attachment centered on the medial epicondyle. Their biomechanical testing showed that a shorter sMCL construct (with a more proximal tibial attachment) was more potent than a longer sMCL construct at controlling ER. However, similar to this study, they found that the addition of an AM limb to the sMCL reconstruction gave increased control of ER in deeper flexion. We also found that an isolated short sMCL construct afforded less reduction of ATT and ER as compared with sMCL + AM reconstructions at 60° and 90° of flexion, although after a short sMCL reconstruction, laxity was not significantly different from the intact state. It must be noted that the short sMCL construct in our study utilized a tibial attachment that was slightly more posterior, positioned in the line of the posterior fibers of the sMCL, as compared with that described by Shatrov et al, which was placed a little more anterior, in line with the midpoint of the anterior 50% of the native sMCL fibers and 20 mm below the joint line. This more oblique nature of the reconstruction tested by Shatrov et al might therefore have been better at controlling rotational stability in higher degrees of flexion when compared with the short sMCL that we tested, which was aligned more vertically and in line with the central fibers of the native sMCL. The difference in efficacy might also be explained by the different material properties of the grafts used in the 2 studies. In our study, an autologous ST autograft was used, whereas synthetic polyester tape was used by Shatrov et al. In addition, there were other methodological differences. We found that cutting the dMCL and sMCL resulted in increased ATT laxity with the AM drawer test, whereas Shatrov et al found no difference at any degree of flexion.

The use of synthetic grafts has been advocated by some in the acute scenario to protect (“brace”) the MCL, allowing it to heal without elongation.^5,12,13,54^ Yet, there is limited evidence on the long-term clinical outcomes of synthetic graft materials used for the MCL, with only short-term results available.^14^ Soft tissue tendon autografts and allografts, however, are not only commonly used to address MCL deficiencies but have long-term follow-up,^10,11,25^ and their usage reflects contemporary expert consensus opinion.^16^

Our study suggests that the addition of an AM reconstruction to an sMCL reconstruction offers optimal control of ER, ATT, and VR. The tibial attachment of the AM reconstruction should be anterior to the most anterior fibers of the sMCL, consistent with previous studies,^4,9^ providing an oblique graft orientation that mimics the course of the anterior fibers of the dMCL. Previous biomechanics studies have suggested that this more oblique orientation of the AM reconstruction is advantageous in restraining ER.^4^ However, our data support previous work that investigated the length change pattern of AM reconstructions^9^ and found that the tibial attachment site of the AM graft is less important than the femoral attachment site in determining graft behavior.^9,36^ This finding is similar to that found for anterolateral reconstructions.^34,35^ Our data also suggested that surgeons should be cautious not to overtension the AM limb of the graft, particularly with the T1 and T2 attachments, as this might lead to some overconstraint.

The modified Lind reconstruction restored ATT and ER similar to the intact state, only failing to completely control VR in 60° and 90° of flexion, where it failed to restore native kinematics. However, this reconstruction leaves the ST tendon attached to its tibial insertion site; thus, the reconstruction requires fewer implants than the T3-based sMCL + AM reconstruction and is potentially advantageous in terms of cost and graft healing at the tibial attachment. Additionally, there was no significant difference between these 2 reconstructions in our biomechanical testing.

Our findings indicate that the tibial attachment of AM reconstructions may be placed in an area anterior to the anterior-most sMCL fibers, between the anterior tibial attachment of the dMCL and the ST insertion, with minimal effect on kinematics. This flexibility allows the tibial attachment of the AM limb of the graft to be placed to avoid conflict with an ACL tibial tunnel without adversely affecting graft performance.

This study has several limitations. The age and quality of the specimens may have adversely affected reliable fixation of the multiple reconstruction techniques performed throughout the testing process. This limitation was partially mitigated by employing double-fixation techniques. The methodological limitation of femoral tunnel expansion during testing is addressed by an already-described technique, in which an interference screw is inserted into the tunnel so that the graft is positioned at the 12-o’clock position against it.^8^ While our results are unlikely to have been affected by the different bone densities at the T1, T2, and T3 tibial attachment sites, owing to the AM grafts being passed transosseously and tensioned on the lateral side, variation in bone density may have implications for choice of fixation in the clinical scenario. We used the same tendon for consecutive reconstruction, and to mitigate against the risk of tendon graft degradation affecting the results, the order of the reconstructions was randomized. However, it was necessary to always test the modified Lind technique first, as this technique leaves the ST tendon attached to its tibial insertion site. This limitation might have led to a relatively better performance for the Lind reconstruction technique as compared with subsequent reconstructions. We also saw no macroscopic deterioration of the tendons and no apparent detrimental effect on the reconstructions throughout testing. Our study was a time-zero analysis and may overlook the effects of ligament tension loss through fixation slippage or stretching during graft healing; therefore, the clinical effect of a potential overconstraint of the reconstructions using the T1, T2, and T3 positions cannot be predicted.

We tested only reconstructions that used a single femoral tunnel. Two- and even 3-bundle MCL reconstructions have been proposed, with separate femoral attachments for each graft; however, previous studies^9^ have recommended a single femoral attachment for surgical simplicity. While separate femoral and tibial tunnels might better replicate the dMCL and sMCL “anatomically,” the close proximity of tunnels around the femoral epicondyle might compromise graft fixation and/or result in tunnel coalition. The optimum position of a single femoral tunnel has been studied with regard to graft length change behavior^9^; thus, we elected to use this for the current study. We also tested only a short single-bundle sMCL reconstruction because an isolated long sMCL reconstruction was previously found to be incapable of restoring native ER laxity.^8^ Similarly, we did not assess an isolated oblique AM reconstruction (ie, without an sMCL reconstruction), as the obliquity is likely to be less effective in the restraint of VR laxity.^13,47^ We also standardized the sMCL reconstruction that we used for assessing the modified Lind and the double-bundle (sMCL + AM) graft constructs, using tibial attachment sites for sMCL reconstruction similar to that described by LaPrade and Wijdicks.^37^ Furthermore, we tested only soft tissue autografts and not synthetic materials.

The effect of the POL was not investigated.^8^ Although the POL significantly contributes to valgus restraint in full extension, it does not play a significant role in AMRI.^28,46,53^ In addition, the dMCL and sMCL, rather than the POL, are much more frequently injured in combination with ACL injuries.^17,54^ However, we did not assess the AM retinaculum, which also contributes to AM stability.^28^ Neither did we assess the role of the medial hamstring muscles as dynamic stabilizers of AMRI. Forces mediated by the medial hamstrings play a role in controlling tibial ER,^27,33^ although this is more important at deeper flexion angles and not significant in the extended knee.^27,33^

This study represents only kinematic data. A potential effect of the biomechanical difference between the modified Lind and double-bundle reconstructions now needs further clinical investigation. Also, we did not investigate the influence of the medial reconstructions on resulting ACL stress or medial contact pressures. This could be the subject of future studies. In particular, increased compartment pressure is associated with an increased risk of the development of osteoarthritis, especially when applied nonisometrically.^21,29,30,43,45^

## Conclusion

AMRI appears to be better restrained by a double-bundle reconstruction replicating the function of the sMCL and dMCL. The tibial attachment of the AM limb of the reconstruction should be anterior to the sMCL, but its precise location on the tibia was found to be less important. This offers surgical flexibility, which may be helpful in avoiding coalition with the ACL tibial tunnel. The attachment site of the femur on the posterior medial epicondyle is critical to optimize graft isometry.
